# Cisplatin induced cerebral sinus venous thrombosis in cervical cancer patients treated with concurrent chemoradiation: a case series

**DOI:** 10.3332/ecancer.2021.1320

**Published:** 2021-11-18

**Authors:** Deepthi Valiyaveettil, Swapna Jilla, Jonnalagadda Mohan Krishna, Raja Kollu, Chandrasekhar Patil, Ranadheer Gupta

**Affiliations:** 1 Department of Radiation Oncology, Malla Reddy Cancer Hospital and Research Institute, Hyderabad 500055, India; 2 Department of Neurology, Malla Reddy Narayana Multispeciality Hospital, Hyderabad 500055, India; 3 Department of Radiology, Malla Reddy Narayana Multispeciality Hospital, Hyderabad 500055, India; 4 Department of Nuclear Medicine, Malla Reddy Cancer Hospital and Research Institute, Hyderabad 500055, India

**Keywords:** carcinoma cervix, cisplatin, radiation, CSVT

## Abstract

Cisplatin is a widely used chemotherapeutic agent. Concurrent chemotherapy with cisplatin is an important component in the management of carcinoma cervix. The common side effects of cisplatin chemotherapy include nausea, vomiting, dyselectrolytemia, nephrotoxicity, etc. These side effects are anticipated and managed during chemotherapy. Thromboembolic events are rare complications with cisplatin. We present three cases of cisplatin related cerebral sinus venous thrombosis (CSVT). These patients were receiving concurrent chemoradiation for carcinoma cervix. These patients presented with neurological symptoms and were evaluated and diagnosed with CSVT. They recovered after appropriate management. Clinicians should be aware that CSVT, though uncommon, is a life threatening complication during cisplatin chemotherapy which should be appropriately evaluated and effectively managed.

## Introduction

Cisplatin is one of the most potent alkylating antineoplastic agents [1] which is widely used for the treatment of several malignancies including cervical cancer [2–5]. Concurrent chemotherapy with cisplatin during radiation has shown better local control and overall survival compared to only radiation in cervical cancer trials [6, 7].

Cisplatin has been linked to various toxic side effects including nausea, nephrotoxicity, cardiotoxicity, hepatotoxicity and neurotoxicity [8]. Renal and gastric toxicities are the most common and anticipated side effects of cisplatin. Thromboembolic events (TEEs) is not a well known toxicity associated with cisplatin but has been reported in the literature. A study reported that 18.1% of cisplatin-treated cancer patients developed TEEs [9].

We present three patients who developed cerebral sinus venous thrombosis (CSVT) while on concurrent chemotherapy with cisplatin during the treatment for cervical cancer.

## Case summaries

### Summary 1

**Figure 1.**
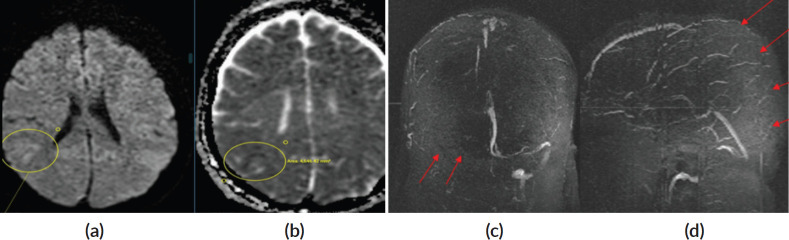
(a and b): Diffusion-weighted imaging (DWI) and Apparent Diffusion coefficient (ADC) images showing diffusion restriction with corresponding low ADC (marked with circle) in right parietal lobe suggesting acute infarction. (c): Non-contrast 3D time of flight (TOF) MR venogram depicts non-visualisation of right transverse and sigmoid sinus – Suggestive of complete thrombosis (red arrows). (d): Non-contrast 3D TOF MR venogram depicts non-visualisation of posterior two third superior sagittal sinus – Suggestive of complete thrombosis (red arrows).

A 49-year-old female patient with no known co-morbidities was diagnosed with carcinoma cervix stage IIB. She received external beam radiotherapy to pelvis along with three cycles of concurrent chemotherapy with cisplatin at 40 mg/m^2^. After 12 days of last chemotherapy, she presented to the emergency department with complaints of fall at home, tongue bite and loss of consciousness. She was evaluated with Computed Tomography (CT) of the brain which showed hyperdensity along superior sagittal and right transverse venous sinuses suggesting the likely diagnosis of CSVT. The Magnetic Resonance Imaging (MRI) of the brain showed thromboses in posterior half of superior sagittal sinus, right transverse and sigmoid sinuses (Figure 1). She was managed with anticoagulants, antiepileptics, analgesics and intravenous fluids. She gradually improved and was discharged in stable condition. Post recovery she completed her planned intracavitary brachytherapy. She is on regular follow-up since 1.5 years with complete response.

### Summary 2

A 37-year-old female with no known co-morbidities was diagnosed with carcinoma cervix stage IVA. She received external beam radiotherapy to pelvis along with four cycles of concurrent chemotherapy with cisplatin at 40 mg/m^2^. She developed right hemiparesis, 9 days after last cycle of cisplatin. On evaluation, the MRI of the brain showed thrombosis in posterior part of superior sagittal sinus, right transverse and sigmoid sinus. She was managed with anticoagulants, antiepileptics and intravenous fluids. She improved clinically and was discharged. Post recovery she completed the remaining course of radiation treatment. Now she is on regular follow-up since 1.5 years with complete response.

### Summary 3

**Figure 2.**
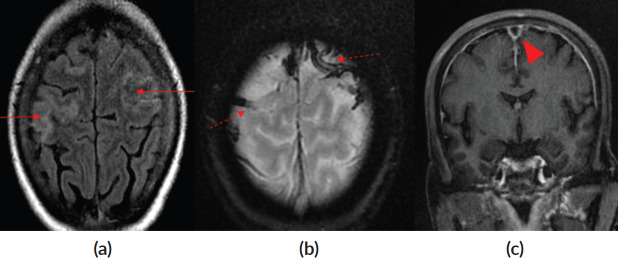
(a): Axial Fluid Attenuated Inversion Recovery (FLAIR) image depicting mild gyral oedema in bilateral high frontoparietal lobes (red arrows). (b): Gradient Recalled Echo (GRE) image showing blooming in cortical veins in bilateral high frontal lobes (red dashed arrows). (c): Post contrast T1 coronal image showing filling defect in superior sagittal sinus suggesting thrombosis (red arrow head).

A 47-year-old female with no known co-morbidities was diagnosed with carcinoma cervix stage IIB. She received external beam radiotherapy to pelvis along with four cycles of concurrent chemotherapy with cisplatin at 40 mg/m^2^. During the course of radiation (post 7 days of fourth cycle chemotherapy), she presented to the hospital with complaints of right upper limb weakness. She was evaluated with the MRI of the brain which showed anterior superior sagittal sinus and bilateral superficial cortical venous thrombosis along with subarachnoid haemorrhage (Figure 2). She was managed with anticoagulants, antiepileptics and intravenous fluids. She recovered clinically and completed planned radiation treatment. She is on follow-up and disease-free since 1 year.

## Discussion

These patients presented with neurological symptoms while on concurrent chemoradiation for cervical cancer. They were evaluated and diagnosed with CSVT early and were managed appropriately. The patients improved clinically post treatment and completed the standard treatment for cervical cancer as planned. All the three patients had no co-morbidities and no prior history of thrombosis. Evaluation for other systemic causes was negative. The time interval between chemotherapy and CSVT among these cancer patients suggests a cisplatin related cause.

Cancer and cisplatin chemotherapy are well-recognised risk factors for coagulation disorders and thrombosis [10]. Cancer is associated with an increased risk of venous and arterial TEEs. These events include deep venous thrombosis, pulmonary embolism, cerebrovascular accident and unstable angina/myocardial infarction. On an average the annual incidence rate of venous TEE in general population is approximately 117 per 100,000, whereas the incidence in cancer patients is around one in 200 [11, 12]. A large cohort study reported cancer alone increases the risk of TEE by 4.1 times and addition of chemotherapy by 6.5 times [13].

CSVT during chemotherapy is rare and only few cases are reported in literature [10]. This condition is less common than other types of stroke. It commonly affects large sinuses like superior sagittal sinus. Mostly no underlying cause is identified. Around 30% of cases are attributed to inherited and systemic inflammatory diseases. The International Study on Cerebral Venous and Dural Sinuses Thrombosis reported 7.4% of cases of CSVT were associated with cancer [14]. It is more common in females with female to male ratio of 3:1.

Antineoplastic drugs like L-asparaginase and tamoxifen are established risk factors for CSVT [15]. Other treatment regimens with increased risk of CSVT are (i) Folinic acid, 5fluorouracil and Irinotecan (FOLFIRI) regimen/bevacizumab regimen in colon cancer management [16], (ii) concurrent chemoradiation with temozolomide and bevacizumab in brain tumour management [17] and (iii) cisplatin, ifosfamide, adriamycin and vincristine regimen in Ewing sarcoma management [18]. CSVT caused by micrometastases from cutaneous melanoma has also been reported [19].

The mechanism for cisplatin-induced coagulopathy is thought to be endothelial injury activating the coagulation cascade and resulting in TEEs [20]. Degenerative processes of vessel walls are initiated which eventually causes occlusive vascular disease. There is evidence regarding excess of myocardial infarctions, arterial hypertension and cerebral strokes in these patients. These complications have been reported in several malignancies but germ cell tumours on cisplatin based regimens are at higher risk. Serum lactate dehydrogenase levels and the body surface area are important risk predictors in these patients [21]. Anticoagulation, treating the underlying cause, controlling the intracranial haemorrhage, antiseizure medication and management of focal deficits are the main treatment strategies [22].

A study observed early occurrence of cardiovascular complications secondary to cisplatin-based chemotherapy. These complications may occur during the chemotherapy cycles or immediately after it. Karam and Koussa [10] reported two cases who presented with cerebral dural sinus thrombosis while on cisplatin based chemotherapy regimen. These patients developed neurological symptoms while on chemotherapy. MRI and Magnetic Resonance Angiography (MRA) brain findings in these patients were suggestive of CSVT. They were managed with anticoagulants and supportive care. Evaluation for other causes of CSVT was negative. They concluded that the development of CSVT in these patients was chemotherapy related. Yamada *et al* [23] reported a case of superior sagittal sinus thrombosis in a 5-year-old girl treated with cisplatin and etoposide regimen for a suprasellar germ-cell tumour. The patient presented with symptoms post two cycles of chemotherapy.

A large retrospective analysis [9], done by Memorial Sloan-Kettering Cancer Center confirms the incidence of TEEs in patients receiving cisplatin-based chemotherapy. They included 932 patients with various cancers. They included 39 (4.2%) patients who had uterine/cervical or vulvar cancers. TEE was reported in 169 (18.1%) patients during treatment or within 4 weeks of the last dose. They concluded that unacceptably high incidence of TEEs (18.1%) is observed during the cisplatin-based chemotherapy for a variety of cancers during the period of administration or within 4 weeks of completion of treatment. They also suggested that TEE prophylaxis may be advisable for patients receiving cisplatin-based chemotherapy.

CSVT was reported in two patients who were on cisplatin based chemotherapy for germ cell tumours [24]. The authors suggested a cisplatin related hypercoagulability as a leading risk factor in both these cases as other causes for cancer related TEEs were ruled out. In one patient, they replaced cisplatin with carboplatin in the third cycle of chemotherapy. The patient again developed CSVT post carboplatin. This further confirms platin based complication.

## Conclusion

Cisplatin is a common and essential antineoplastic drug in the management of several malignancies. CSVT as a complication of cisplatin chemotherapy is uncommon and alarming. Clinicians should be aware of the potential risk of development of this neurological side effect. Early diagnosis and appropriate treatment are necessary for complete recovery.

## Conflicts of interest

The authors declare that they have no conflicts of interest.

## Funding source

Nil.

## References

[1] Dasari S, Tchounwou PB (2014). Cisplatin in cancer therapy: molecular mechanisms of action. Eur J Pharmacol.

[2] Ghosh S (2019). Cisplatin: the first metal based anticancer drug. Bioorg Chem.

[3] Pignon JP, Tribodet H, Scagliotti GV (2008). Lung adjuvant cisplatin evaluation: a pooled analysis by the LACE Collaborative Group. J Clin Oncol.

[4] Hu XC, Zhang J, Xu BH (2015). Cisplatin plus gemcitabine versus paclitaxel plus gemcitabine as first-line therapy for metastatic triple-negative breast cancer (CBCSG006): a randomised, open-label, multicentre, phase 3 trial. Lancet Oncol.

[5] Cooper JS, Pajak TF, Forastiere AA (2005). Postoperative concurrent radiotherapy and chemotherapy for high-risk squamous-cell carcinoma of the head and neck. N Engl J Med.

[6] Eifel PJ, Winter K, Morris M (2004). Pelvic irradiation with concurrent chemotherapy versus pelvic and para-aortic irradiation for high-risk cervical cancer: an update of radiation therapy oncology group trial (RTOG) 90-01. J Clin Oncol.

[7] Morris M, Eifel PJ, Lu J (1999). Pelvic radiation with concurrent chemotherapy compared with pelvic and para-aortic radiation for high-risk cervical cancer. N Engl J Med.

[8] Singh L, Aldosary S, Saeedan AS (2018). Prolyl hydroxylase 2: a promising target to inhibit hypoxia-induced cellular metabolism in cancer cells. Drug Discov Today.

[9] Moore RA, Adel N, Riedel E (2011). High incidence of thromboembolic events in patients treated with cisplatin-based chemotherapy: a large retrospective analysis. J Clin Oncol.

[10] Karam C, Koussa S (2008). Cerebral dural sinus thrombosis following cisplatin chemotherapy. J Clin Neurosci.

[11] Silverstein MD, Heit JA, Mohr DN (1998). Trends in the incidence of deep vein thrombosis and pulmonary embolism: a 25-year population-based study. Arch Intern Med.

[12] Lee AY, Levine MN (2003). Venous thromboembolism and cancer: risks and outcomes. Circulation.

[13] Heit JA, Silverstein MD, Mohr DN (2000). Risk factors for deep vein thrombosis and pulmonary embolism: a population-based case-control study. Arch Intern Med.

[14] Ferro JM, Canhão P, Stam J (2004). Prognosis of cerebral vein and dural sinus thrombosis: results of the international study on cerebral vein and dural sinus thrombosis (ISCVT). Stroke.

[15] Saposnik G, Barinagarrementeria F, Brown Jr RD (2011). Diagnosis and management of cerebral venous thrombosis: a statement for healthcare professionals from the American Heart Association/American Stroke Association. Stroke.

[16] Ozen A, Cicin I, Sezer A (2009). Dural sinus vein thrombosis in a patient with colon cancer treated with FOLFIRI/bevacizumab. J Cancer Res Ther.

[17] Vargo JA, Snelling BM, Ghareeb ER (2011). Dural venous sinus thrombosis in anaplastic astrocytoma following concurrent temozolomide and focal brain radiotherapy plus bevacizumab. J Neurooncol.

[18] Unal E, Yazar A, Koksal Y (2008). Cerebral venous sinus thrombosis in an adolescent with Ewing sarcoma. Childs Nerv Syst.

[19] Basiri K, Dashti M, Fatehi F (2010). Malignant cutaneous melanoma associated with cerebral venous sinus thrombosis. Neurosciences (Riyadh).

[20] Ito H, Okafuji T, Suzuki T Vitamin E prevents endothelial injury associated with cisplatin injection into the superior mesenteric artery of rats. Heart Vessels.

[21] Piketty AC, Fléchon A, Laplanche A (2005). The risk of thrombo-embolic events is increased in patients with germ-cell tumours and can be predicted by serum lactate dehydrogenase and body surface area. Br J Cancer.

[22] Alvis-Miranda HH, Castellar-Leones SM, Alcala-Cerra G (2013). Cerebral sinus venous thrombosis. J Neurosci Rural Pract.

[23] Yamada K, Yamashiro S, Itoyama Y (1993). Sinus thrombosis during CDDP and VP-16 (PE) therapy for suprasellar germ-cell tumor: case report. No Shinkei Geka.

[24] Papet C, Gutzeit A, Pless M (2011). Two cases of cerebral sinus venous thrombosis following chemotherapy for non-seminomatous germ cell tumor. Case Rep Oncol.

